# Kinematic characterization of clinically observed aberrant movement patterns in patients with non-specific low back pain: a cross-sectional study

**DOI:** 10.1186/s12891-017-1820-x

**Published:** 2017-11-15

**Authors:** Peemongkon Wattananon, David Ebaugh, Scott A. Biely, Susan S. Smith, Gregory E. Hicks, Sheri P. Silfies

**Affiliations:** 1 Motor Control and Neural Plasticity Laboratory, Faculty of Physical Therapy, Mahidol University, 999 Phuttamonthon 4 Road, Salaya, Nakhon Pathom, 73170 Thailand; 2 Physical Therapy & Rehabilitation Sciences Department, Drexel University, 1601 Cherry Street, Philadelphia, PA 19102 USA; 3 Physical Therapy Program, Neumann University, One Neumann Drive, Aston, PA 1901 USA; 4 College of Nursing and Health Professions, Drexel University, 245 N 15th St, Philadelphia, PA 19102 USA; 5 Department of Physical Therapy, University of Delaware, 540 S. College Ave, Suite 210E, Newark, DE 19713 USA

**Keywords:** Low back pain, Multi-segment kinematics, Clinical observation, Aberrant movement patterns

## Abstract

**Background:**

Clinical observation of aberrant movement patterns during active forward bending is one criterion used to identify patients with non-specific low back pain suspected to have movement coordination impairment. The purpose of this study was to describe and quantify kinematic patterns of the pelvis and trunk using a dynamics systems approach, and determine agreement between clinical observation and kinematic classification.

**Method:**

Ninety-eight subjects performed repeated forward bending with clinical observation and kinematic data simultaneously collected. Kinematic data were plotted using angle-angle, coupling-angle, and phase-plane diagrams. Accuracy statistics in conjunction with receiver operating characteristic curves were used to determine agreement between clinical observation and kinematic patterns.

**Results:**

Kinematic patterns were consistent with clinical observation and definitions of typical and aberrant movement patterns with moderate agreement (kappa = 0.46–0.50; PABAK = 0.49–0.73). Early pelvic motion dominance in lumbopelvic coupling-angle diagram ≥59° within the first 38% of the movement represent observed altered lumbopelvic rhythm. Frequent disruptions in lumbar spine velocity represented by phase-plane diagrams with local minimum occurrences ≥6 and sudden decoupling in lumbopelvic coupling-angle diagrams with sum of local minimum and maximum occurrences ≥15 represent observed judder.

**Conclusion:**

These findings further define observations of movement coordination between the pelvis and lumbar spine for the presence of altered lumbopelvic rhythm and judder. Movement quality of the lumbar spine segment is key to identifying judder. This information will help clinicians better understand and identify aberrant movement patterns in patients with non-specific low back pain.

## Background

Low back pain is one of the most common health problems in the United States [1]. More importantly, high prevalence and recurrence rates increase the number of medical visits, hospitalization, and utilization of health care services, including physical therapy [1–3]. Low back pain that is not attributable to a recognizable or known specific pathology is referred to as a non-specific low back pain (NSLBP). Non-specific low back pain is accountable for approximately 85% of all low back pain [4].

Current clinical research suggests patients with NSLBP demonstrate different clinical characteristics that may result from different underlying contributory mechanisms [5–9]. Impairment in inter-segmental movement coordination (e.g., coordination between the lumbar spine, pelvis and hip) has been proposed as one cause of NSLBP [8, 10]. Movement coordination impairment (MCI) is defined as poorly coordinated or controlled spine and pelvis position and movement during functional tasks that places repeated abnormal stresses on musculoskeletal tissues eventually contributing to tissue injury and pain [11]. Clinicians have assumed that MCI is associated with impaired neuromuscular control that can be identified by clinical observation of aberrant movement patterns [9, 11–17].

Evidence supports that aberrant movement patterns observed during an active forward bending task is one identifier of patients with MCI and that these patients benefit from exercises focused on trunk muscles and designed to improve coordination and control (e.g., core stabilization or lumbar stabilization) [7, 18]. Recent work has demonstrated that clinical observation of aberrant movement patterns during standing forward bend has fair to almost perfect (kappa = 0.35–0.89) inter-rater reliability when motion was observed simultaneously by two experienced clinicians [19]. Findings from this study also revealed that aberrant movement patterns were significantly associated with NSLBP providing construct validity for the association of aberrant movement patterns with current symptoms [19]. Furthermore, a greater frequency of aberrant patterns can be seen in patients with current NSLBP compared to healthy controls when performing multiple repetitions of forward bend [19].

Although evidence supports the use of clinical observation for identifying patients with MCI, investigators have not systematically captured, described, or quantified typical and aberrant movement patterns using continuous kinematic data of multiple body segments (femur, pelvis, lumbar spine, and thoracic spine) during a forward bending motion. As a result, clinicians have limited information about which segments and movement characteristics (range, velocity, and/or timing) significantly contribute to the observed aberrant movement patterns.

Kinematic data have been widely used for investigating the amount of trunk and pelvic motion during forward bending, with limited investigation into the movement patterns and underlying neuromuscular control [20–22]. Kinematics, in conjunction with a dynamic systems approach, can be used to better understand movement patterns [22–25]. By plotting continuous angle changes between different body segments, or continuous angle changes against segmental instantaneous angular velocity, kinematic data can be used to represent patterns of movement (inter-segment coordination, and movement control) during functional motions [26]. The purposes of this study were to 1) describe and quantify temporal and spatial 3-dimensional multi-segmental kinematics of the pelvis and trunk using a dynamics systems approach, and 2) determine agreement between clinical observation and kinematic classification of movement patterns. Detailed kinematic descriptions of these patterns should provide clinicians with the ability to enhance their knowledge and understanding of inter-segment coordination and movement control associated with different aberrant movement patterns observed during forward bending. This could lead to better identification and treatment of MCI, and provide a significant step toward quantification of aberrant movement.

## Methods

### Subjects

Ninety-eight subjects with both clinical observation and kinematic data recorded simultaneously during a series of forward bending tasks were used in this secondary data analysis [19]. Subjects were between 18 and 65 years of age and took part in a study conducted within a university and private physical therapy clinic. This study was approved by the university institutional review board, and all subjects provided written informed consent prior to participation. Thirty-five subjects had no history of LBP, 29 subjects were experiencing a current episode of LBP that started within the past 7 weeks, and 34 had a history of LBP but were currently pain free (Table 1). Exclusion criteria for all subjects consisted of: 1) clinical signs of systemic disease; 2) definitive neurologic signs including weakness or numbness in the lower extremity; 3) previous spinal operation; 4) diagnosed osteoporosis, severe spinal stenosis, and/or inflammatory joint disease; 5) pregnancy; 6) any lower extremity condition that would potentially alter trunk movement in standing; 7) vestibular dysfunction; 8) extreme psychosocial involvement; or 9) active treatment of another medical condition that would preclude participation in any aspect of the study.

**Table 1:** Demographic data for control, current episode of LBP, and history of LBP subjects

	N	%Female	Age ± SD (years)	NPRS ± SD (score0–10)	ODI ± SD (score0–100)
Control	35	57.1	40.9 ± 9.3	N/A	N/A
Current episode of LBP	29	48.3	43.6 ± 12.3	4 ± 2.6	28 ± 14.1
History of LBP	34	50.0	46.7 ± 9.5	N/A	N/A
Total	98	52.0	43.7 ± 10.5	N/A	N/A
Group comparison *p* value	N/A	0.75^a^	0.07^b^	N/A	N/A

*LBP* Low back pain, *NPRS* Numeric pain rating scale, *ODI* Oswestry disability index, *SD* Standard deviation

^a^Group comparison using a chi-square test

^b^Group comparison using a one-way analysis of variance (ANOVA)

### Procedures and kinematic instrumentation

Subjects performed 6 repetitions of an active forward bend task. Two experienced physical therapists observed the forward bend task while kinematic data was simultaneously collected. These therapists had at least 5 years of experience in spinal rehabilitation and completed a 2-h training session that standardized the definitions of aberrant patterns prior to data collection. For each subject, the therapists, who were blinded to the group assignment, independently rated the movement pattern as typical or aberrant. Table 2 provides operational definitions of typical and aberrant movement patterns used by these clinicians to assess movement during standing forward bending [7, 12, 15, 17, 19, 27–29].

**Table 2:** Operational definitions of clinically observed typical and aberrant movement patterns during a standing forward bend and return motion

Movement pattern type	Operational definition
Typical	During the forward bend phase, hip and lumbar spine motion occur simultaneously with lumbar spine motion predominating in the first 1/3^rd^ and hip motion predominating in the last 1/3^rd^ of the movement. During the return to upright phase, hip and lumbar spine motion occur simultaneously with hip motion predominating in the first 1/3^rd^ and lumbar spine motion predominating in the last 1/3^rd^ of the movement. Movement should be smooth (gradual increase and decrease in velocity) and remain in the sagittal plane.
Altered lumbopelvic rhythm (aLPR)	During the forward bend phase, hip motion is greater than lumbar spine motion during the first 1/3^rd^ and/or lumbar motion greater than hip motion during the last 1/3^rd^ of the movement, or during return to an upright position, lumbar spine motion is greater than hip motion during the first 1/3^rd^ and/or hip motion greater than lumbar spine motion during the last 1/3^rd^ of the movement.
Judder (JUD)	Observation of a sudden deceleration and acceleration, or quick out of sagittal plane movements during trunk forward bending or return.

**Fig. 1:**
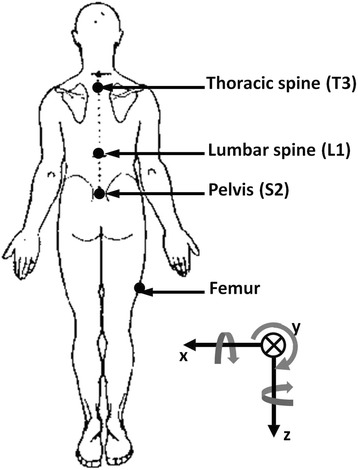
Location of kinematic sensors on the femur, pelvis, lumbar spine, and thoracic spine. Cardan sequence was x (flexion positive), y (right side bend positive), and z (right rotation positive)

An electromagnetic tracking system (3Space Fastrak, Polhemus Inc., Colchester, VT) was utilized to capture position and orientation of thoracic and lumbar spine, pelvis, and thigh segments at 30 Hz during forward bend and return to standing. Kinematic sensors were mounted to orthoplast and attached to the subject at the following body landmarks (Fig. 1): 1) right femur (15 cm. superior to the right femoral lateral epicondyle), 2) pelvis (over the spinous process of S2), 3) lumbar spine (over the spinous process of L1), and 4) thoracic spine (over the spinous process of T3). Based upon the recommendations of the International Society of Biomechanics (ISB), the following anatomical landmarks were digitized to create a local reference frame for each body segment: 1) thorax (sternal notch, xyphoid process, T8, and C7); 2) pelvis (right ASIS, left ASIS, right PSIS, and left PSIS); 3) lumbar spine (xyphoid process, T10, L3-L5); and 4) femur (medial epicondyle, lateral epicondyle, and femoral head) [30].

Preliminary work conducted in our lab established the intra- and inter-session coefficient of multiple correlation (CMC) for measuring movement patterns with the electromagnetic tracking system. The CMC was fair to excellent (intra-session CMC = 0.76–0.95 and inter-session CMC = 0.51–0.95) across segments demonstrating consistency of the movement patterns in standing forward bend for time-series range of motion and angular velocity. The lower CMC values were associated with movements in the frontal plane.

### Data reduction

Mutual agreement on clinical observation by the two experienced clinicians was used for stratification of individual kinematic patterns derived from the forward bending task (98 subjects × 6 repetitions = 588 movement patterns) into typical or aberrant patterns of movement. This stratification was performed independent of the subject’s low back pain status.

Kinematic data reduction was completed using custom LabVIEW programs (National Instruments, Austin, TX.). Data were converted to segmental angular rotations using Euler angles following a Cardan sequence of x (flexion/extension), y (lateral bend to the right/left, and z (rotation to the right/left) (Fig. 1). Segmental rotations included: 1) total trunk motion (FT; thoracic spine motion with respect to the femur); 2) pelvic motion (FP; pelvic motion with respect to the femur); 3) lumbar motion (PL; lumbar spine motion with respect to the pelvis); 4) thoracic motion (LT; thoracic spine motion with respect to the lumbar spine); and 5) thoracolumbar motion (PT; combined lumbar-thoracic spine motion with respect to the pelvis).

Based on total trunk motion, a LabVIEW program was used to determine the start and stop points for each repetition of the forward bend motion. Kinematic data were then filtered with a dual pass Butterworth filter (2nd order low pass frequency at 5 Hz) and time-normalized to 51 data points (0–50) to represent 100% of the forward bend motion. Typical and aberrant movement patterns were represented by the following kinematic diagrams: 1) angle-angle, 2) coupling-angle, and 3) phase-plane diagrams [23, 31–33].

### Kinematic representation and interpretation of movement patterns

#### Inter-segmental coordination

**Fig. 2:**
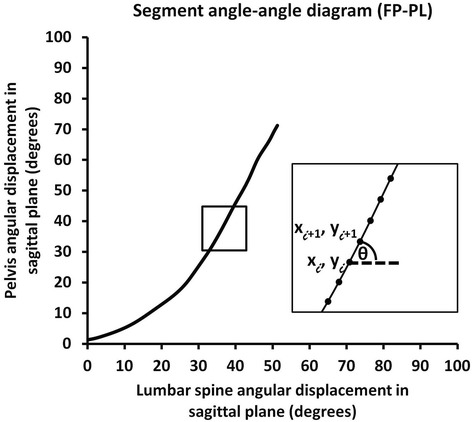
Example of a lumbopelvic (FP-PL) segment angle-angle diagram from one subject. One segment’s angular displacement (x axis) in sagittal plane versus another segment’s angular displacement (y axis) in sagittal plane during forward bend. Altered lumbopelvic rhythm was defined as slope greater than 45° in the first 1/3rd of the movement. To quantify coordination changes observed in a segment angle-angle diagram, a coupling angle (insert), which is the angle between the vector formed between two adjacent data points relative to the right horizontal, *coupling angle (θ) = atan [(Y*_*i + 1*_ *–Y*_*i*_ *)/(X*_*i + 1*_ *–X*_*i*_ *)]*, was used to standardize segment coordination across trials and subjects

**Fig. 3:**
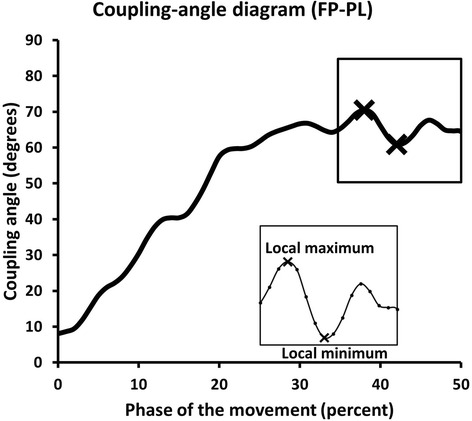
Example of a lumbopelvic (FP-PL) coupling-angle diagram that plots percentage total angular displacement during forward bending (x axis) versus coupling angles (y axis). Relative timing of a shift from lumbar domination to pelvic domination within the movement pattern is defined by % total angular displacement when the coupling angles are greater than 46°. Local minimum (LMin) or maximum (LMax) occurrences (insert) representing coordination changes in the coupling-angle diagrams was identified by the greatest (local maximum) or least (local minimum) values (X)

Coordination of movement between the lumbar spine and pelvis is clinically referred to as lumbopelvic rhythm (LPR). This characteristic of movement can be captured and described using a segment angle-angle diagram (Fig. 2). The shape or trajectory of the diagram provides information regarding qualitative coordination between two segments. A diagonal straight line indicates that the two segments are moving at a constant ratio. Horizontal or vertical lines indicate that one segment is moving, whereas the other segment is not [33]. A limiting factor of using angle-angle diagrams to represent LPR arises when subjects move through different amounts of motion. Vector coding (Fig. 2) can be used to address this limitation by standardizing a segment’s contribution by calculating a vector (coupling-angle) between two adjacent points relative to the right horizontal [31, 34]. A coupling-angle diagram (Fig. 3) also represents coordination between segments, and quantifies the shape or trajectory of movement coordination relative to the percent of movement. A coupling angle of 45° indicates 1:1 motion between segments, greater than 45° indicates distal segment (pelvis) dominance; while less than 45° indicates proximal segment (lumbar spine) dominance [33]. These diagrams were also used to determine when, during the motion (% of movement), one segment dominated the motion relative to another segment.

#### Movement control

**Fig. 4:**
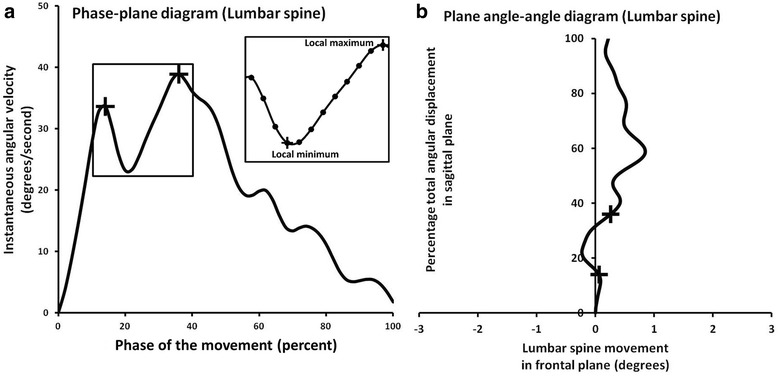
**a** Example of lumbar spine phase-plane diagram representing percentage of total angular displacement (x axis) versus instantaneous angular velocity (y axis) during forward bending. Local minimum and maximum occurrence of the phase-plane diagram (+, insert) represent disruptions in angular velocity (sudden deceleration and acceleration) that are associated with judder. This pattern can also be characterized by quick out of plane deviation in the pelvis or lumbar spine plane angle-angle diagram (**b**) over a short period of time (as indicated by +, in both diagrams). These out of plane deviations are consistent with sudden decreases and increases in angular velocity in the pelvis or lumbar spine phase plane diagram

Movement control of a body segment was captured and described using phase-plane and plane angle-angle diagrams (Fig. 4). Movement control is characterized by smoothness of the segment’s velocity. Disruptions in control can be identified by the number of local minimum (LMin) and maximum (LMax) occurrences [23]. These occurrences represent sudden deceleration and acceleration during movement tasks that are clinically referred to as judder (JUD). Quick out of sagittal plane movement or off axis motion is another sign of impaired control that is another focus of clinical definition of judder. This presentation of poor movement control can also be captured and described using plane angle-angle and phase-plane diagrams. Additionally, changes in movement control of one segment might cause changes in the relative coordination between segments. Therefore, phase-plane, plane angle-angle, and coupling-angle diagrams can be used to detect the segment responsible for coordination changes identified in a coupling-angle diagram.

### Statistical analysis

For the first purpose of this study, temporal and spatial 3-dimensional kinematics of the pelvis and trunk segments (lumbar, thoracic) associated with both typical and aberrant forward bend movement patterns were described using means and standard deviations of derived kinematic variables (Table 3). The kinematic data were also graphed and additional descriptors were developed.

**Table 3:** Kinematic variables used to describe aberrant movement pattern

Aberrant movement pattern	Variable
aLPR	Slope of AA (Mean CA), Timing
JUD	LMin, LMax

*aLPR* Altered lumbopelvic rhythm, *JUD* Judder, *AA* Angle-angle diagram, *CA* Coupling angle diagram, *Timing* When in the movement aLPR occurred, *LMin* Local minimum occurrence, *LMax* Local maximum occurrence

Individual kinematic variables were tested for normality and homogeneity of variance assumptions. Independent t-test (1-tailed) was used to test for differences between typical and aberrant movement patterns when those assumptions were met, while Mann-Whitney U test was used if those assumptions were violated. We intended to initially remove kinematic variables that did not differentiate between typical and aberrant movement patterns, but we did not wish to exclude any potentially useful kinematic variables. Therefore, we decided to use a liberal approach, in which individual kinematic variables with *p*-value less than 0.10 (*p* < 0.10) were retained as potential key variables of segment and movement characteristics that would then be used to determine agreement between clinical observation and kinematic classification. Additionally, the mean and standard deviation for each kinematic data point from typical movement patterns were used to generate a mean typical movement pattern along with standard deviation bands that represented typical movement variability. Aberrant movement patterns were then plotted against these typical patterns to further describe differences in movement quality. We found that the derived angle and velocity changes at the start and end of motion often caused errors in coupling-angle and phase-plane diagrams secondary to significant variability associated with values fluctuating around zero angular motion or velocity. Therefore, we used data between 5% and 95% of total trunk motion for further analysis.

The second purpose of this study was to determine the level of agreement between clinical observation and kinematic classification derived from kinematic diagrams that represent segments and movement characteristics contributing to the clinically observed aberrant movement patterns. An accuracy statistics approach in conjunction with receiver operating characteristic curves (ROC) was used for this analysis [35–38]. For altered lumbopelvic rhythm (aLPR, inter-segment coordination), we did not know at which point in the movement pattern, clinicians perceived onset of pelvic domination (pelvic-dominated angle). Therefore, we varied the lumbopelvic coupling angle from 45°-90°, then derived a variable, called “timing” for each 1° pelvic-dominated angle increase, to determine when during the motion (% of forwarding bending movement) the pelvis dominated the motion relative to the lumbar spine.

For judder (smoothness of movement), local minimum (LMin) and local maximum (LMax) occurrences in the phase-plane diagrams for each segment (FT, FP, PL, LT, and PT) and coupling-angle diagram for FP-PL were used to quantify movement control and inter-segment coordination, respectively. LMin and LMax in the phase-plane and coupling-angle diagrams correspond to the two operational definitions of judder (sudden deceleration and acceleration, and quick off axis or out of plane movement). However, clinical observation data did not indicate what type of judder had been identified. Therefore, after key segment and characteristics were identified, we further classified judder into quick out of plane movement based on corresponding plane angle-angle diagrams and calculated prevalence of this type of judder.

Contingency tables and receiver operating characteristic curves (ROC) were created using the total number of typical and each aberrant movement pattern based on clinical observation (reference standard) and the kinematic variables derived from quantification of kinematic diagrams. The pelvic-dominated angle and segment that generated the optimal area under the ROC curve (AUC) were then identified. The ROC of identified pelvic-dominated angle or segment and its kinematic variable was used to determine the cut-off point that maximized agreement on kinematic variables. Kappa values were used to assess the agreement between clinical observation and kinematic classification. Sensitivity, specificity, and positive and negative likelihood ratios (LR) were calculated for the cut point. Statistical analysis was performed using custom LabVIEW (National Instruments, Austin, TX) and SPSS (IBM SPSS Statistics for Windows, Version 21.0. Armonk, NY) software.

## Results

### Movement pattern classification: Clinical observation

Based upon clinical observation, two experienced clinicians mutually agreed on 195 out of 588 movement trials (33%). One hundred and eight forward bend movement trials (18%) were classified as a typical, 57 trials (10%) were classified as demonstrating altered inter-segment coordination (LPR), and 30 trials (5%) were classified as demonstrating poor movement control (JUD). In most trials, the thoracic segment’s movement pattern did not assist in the identification of any aberrant pattern. Therefore, this segment was not included in statistical analyses related to our second purpose.

### Kinematic classification

#### Inter-segment coordination

The overall slope of the lumbopelvic (FP-PL) angle-angle diagram for those patterns with aLPR was significantly steeper than that of the typical movement pattern (Table 4). When broken down into specific ranges of the forward bend motion, the aLPR angle-angle slopes in the first and second third of the motion were significantly steeper than typical; however, in the last third of the motion, aLPR slopes were significantly less steep than typical.

**Table 4:** Mean and standard deviation of slope of typical and altered lumbopelvic rhythm (aLPR) angle-angle diagram (mean coupling angle) of lumbopelvic segments (FP-PL) for the first, second, and last 1/3rd of motion, and overall motion

Segment	Group	Slope			
		First 1/3	Second 1/3	Last 1/3	Overall
FP-PL	Typical aLPR	32.0 ± 16.6* 44.7 ± 17.5	51.3 ± 11.0* 63.0 ± 10.4	69.17 ± 18.1* 58.91 ± 15.7	51.99 ± 7.0* 60.26 ± 7.4

* = statistical significance (*p* < 0.10)

Analysis of the coupling-angle diagram revealed that the time (% of movement) when the pelvic contribution was greater than the lumbar contribution occurred significantly earlier in the aLPR patterns (21.9 ± 14.1) when compared with the typical pattern (30.6 ± 16.5). This variable was retained for analysis of agreement between clinical observation and kinematic classification of aLPR.

#### Movement control

The numbers of LMin, and LMax occurrences, as well as the sum of local minimum and maximum (LSum) occurrences on the phase-plane and coupling-angle diagrams were significantly greater for JUD than the typical patterns (Tables 5 and 6). These kinematic variables were retained to determine the agreement between clinical observation and kinematic classification of judder.

**Table 5:** Mean and standard deviation local minimum (LMin), local maximum (LMax), and sum of local minimum and maximum (LSum) occurrences of clinically observed typical and judder (JUD) patterns using phase-plane diagram for each segment

Segment	Group	LMin	LMax	LSum
FT	Typical JUD	2.7 ± 1.4* 5.1 ± 2.4	3.6 ± 1.4* 5.9 ± 2.2	6.3 ± 2.7* 11.0 ± 4.5
FP	Typical JUD	2.1 ± 1.4* 5.5 ± 3.0	4.1 ± 1.3* 6.1 ± 2.7	7.3 ± 2.6* 11.6 ± 5.7
PL	Typical JUD	3.0 ± 1.3* 5.6 ± 2.5	3.7 ± 1.2* 6.1 ± 2.2	6.7 ± 2.5* 11.7 ± 4.6
PT	Typical JUD	1.3 ± 1.0* 2.6 ± 1.5	1.9 ± 0.9* 3.1 ± 1.2	3.2 ± 1.9* 5.7 ± 2.6

*FT* Total trunk (Thoracic spine (T_3_) with respect to right femur), *FP* Pelvic segment (Pelvis (S_2_) with respect to right femur), *PL* Lumbar segment (Lumbar spine (L_1_) with respect to pelvis (S_2_)), *PT* Thoracolumbar segment (Thoracic spine (T_3_) with respect to pelvis (S_2_))

* = statistical significance (*p* < 0.10)

**Table 6:** Mean and standard deviation of local minimum (LMin), local maximum (LMax), and sum of local minimum and maximum (LSum) occurrences of clinically observed typical and judder (JUD) using the lumbopelvic (FP-PL) coupling-angle diagram

Segment	Group	LMin	LMax	LSum
FP-PL	Typical JUD	3.6 ± 1.4* 5.5 ± 3.3	4.3 ± 1.3* 6.3 ± 3.3	8.0 ± 2.7* 11.8 ± 6.7

* = statistical significance (*p* < 0.10)

### Clinical and kinematic agreement

**Fig. 5:**
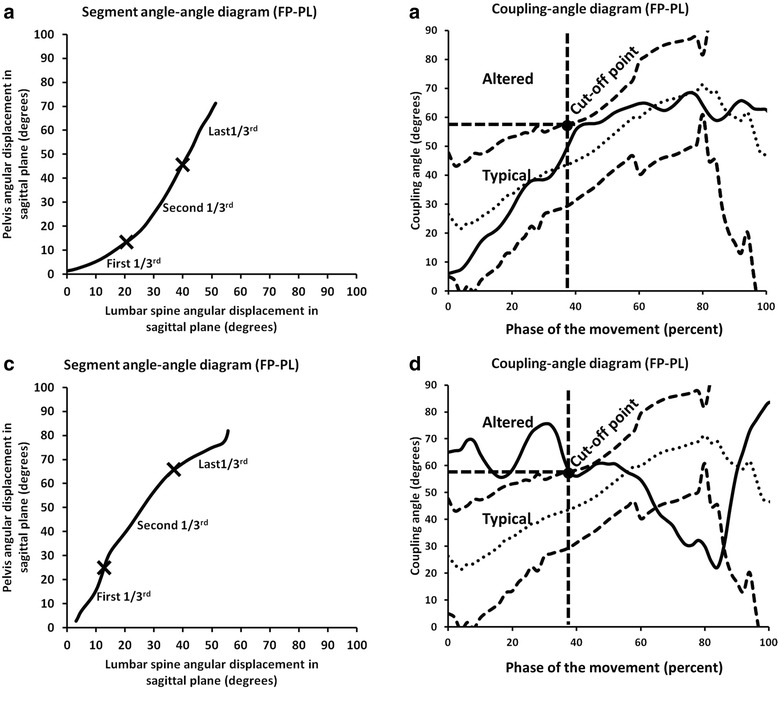
Example of typical lumbopelvic rhythm in a lumbopelvic (FP-PL) angle-angle diagram (**a**) and a lumbopelvic (FP-PL) coupling-angle diagram (**b**), and an example of altered lumbopelvic rhythm in a lumbopelvic (FP-PL) angle-angle diagram (**c**), and a lumbopelvic (FP-PL) coupling-angle diagram (**d**). Solid line represents individual pattern, dotted line represents mean typical pattern, and dashed lines represent ±1 standard deviation of the typical pattern. “X”s are placed to divide total movement into first, second and last 1/3rd of the movement in the angle-angle diagram (**a** and **c**). Typical angle-angle diagram demonstrates the sequence of lumbar spine domination, shared, then pelvis domination; whereas altered lumbopelvic rhythm demonstrates a reversed sequence as pelvis domination in first 2/3rd, then lumbar spine domination (**a** and **c**). In coupling-angle diagram (**b** and **d**), altered lumbopelvic rhythm pattern (**d**) is identified if it falls in altered area (coupling angle greater than 59° before completion of 38% of the forward bend)

**Fig. 6:**
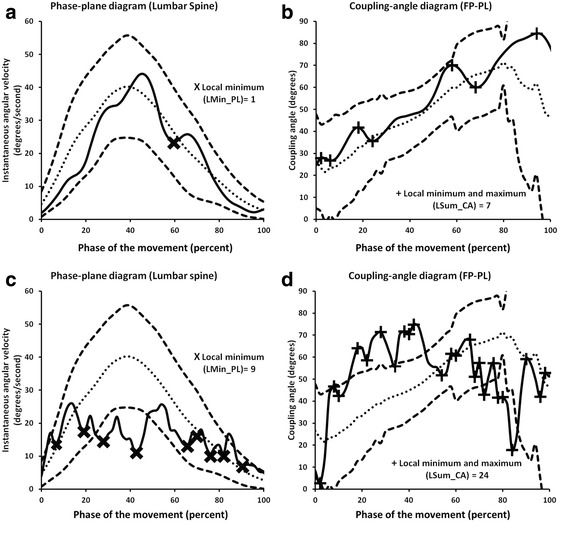
Example of typical movement pattern in a lumbar phase-plane diagram (**a**) with local minimum occurrences (X) and a lumbopelvic (FP-PL) coupling angle (**b**) with local minimum and maximum occurrences (+), and example of judder in a lumbar phase-plane diagram (**c**) with local minimum occurrences (X) and a lumbopelvic (FP-PL) coupling angle diagram (**d**) with local minimum and maximum occurrences (+). Solid line represents individual pattern, dotted line represents mean typical pattern, and dashed lines represent ±1 standard deviation of the typical pattern

Accuracy statistics and ROC analysis revealed that lumbar spine segment kinematics were the key for separating typical from aberrant movement patterns. Table 7 shows the kinematic variables used for classification, AUC, kappa, and accuracy statistics at ROC cut-off point for each aberrant pattern. In addition, we found that 7 out of 30 judder patterns (23%) were further classified as quick out of plane movement based on lumbar spine plane angle-angle diagram. Figures 5 and 6 demonstrate examples of typical and aberrant patterns along with the kinematic variables used to identify the aberrant motion observed by the clinicians.

**Table 7:** Agreement (95% CI) between clinical observation and kinematic lumbopelvic segment movement characteristics and accuracy statistics of the kinematic variables for predicting the observed movement pattern

Type	Variable	Diagram	AUC	%Agreement	Kappa	PABAK	χ^2^	*p* value	Sensitivity	Specificity	+LR	-LR
aLPR	CA_FP_PL@59 and Timing < 38%	CA	0.73	74.55	0.47 (0.32–0.60)	0.49 (0.33–0.62)	38.33	<0.001	0.60 (0.52–0.67)	0.86 (0.79–0.91)	4.27 (2.49–7.62)	0.46 (0.36–0.61)
JUD	LMin_PL ≥ 6	PP	0.85	85.93	0.50 (0.30–0.59)	0.73 (0.58–0.84)	40.55	<0.001	0.43 (0.30–0.49)	0.98 (0.94–0.99)	22.75 (5.44–143.44)	0.58 (0.51–0.74)
	LSum_CA ≥ 15	CA	0.66	85.19	0.46 (0.26–0.51)	0.71 (0.55–0.82)	36.75	<0.001	0.37 (0.25–0.40)	0.99 (0.96–0.1.00)	38.5 (5.69–796.05)	0.64 (0.60–0.79)

Typical (*N* = 108); Altered lumbopelvic rhythm (aLPR; *N* = 57); Judder (JUD; *N* = 30)

*AUC* Area under the receiver operating characteristic curve, *PABAK* Prevalence-adjusted bias-adjusted kappa, *+LR* Positive likelihood ratio, *−LR* Negative likelihood ratio, *CA* Coupling-angle diagram, *PP* Phase-plane diagram, *CA_FP_PL@59 and Timing < 38%* Coupling angle of pelvis and lumbar spine at 59 degrees cut-off and 38% of movement in coupling angle reached 59 degrees for FP-PL, *LMin_PL* Number of local minimum occurrences (lumbar spine), *LSum_CA* Total number of local minimum and maximum occurrences (lumbopelvic coupling angle)

## Discussion

Our kinematic analysis of forward bend movement patterns provides information that enhances the understanding of pelvis and trunk movement characteristics associated with typical and aberrant movement patterns. Additionally, our findings identified the key segments (pelvis and lumbar spine) and movement characteristics of these segments that best described aberrant movement patterns observed during clinical examination of forward bending. Kinematic classifications representing these segments (Table 7) demonstrated high specificity and positive likelihood ratio, which indicates that our kinematic variables have the ability to detect clinically observed aberrant movement patterns from the kinematic data. Detailed kinematic descriptions of typical and aberrant movement patterns are discussed in detail within the following paragraphs.

### Typical forward bend movement pattern

Inter-segment coordination or lumbopelvic rhythm during a forward bend motion is described as smooth and continuous motion with the first third being dominated by lumbar spine motion, the second third shared motion between the lumbar and pelvic segments, and the last third being dominated by pelvic motion (Table 4). Typical lumbopelvic coordination, represented in an angle-angle diagram, is characterized by a smooth concave line with gradual changes in segmental dominance (Fig. 5a). When plotted in a coupling-angle diagram, typical lumbopelvic rhythm is represented by a diagonal line with a positive slope from lower left corner to upper right corner (Fig. 5b). This kinematic description is consistent with the clinical definition of typical lumbopelvic rhythm described by Calliet and Farfan [27, 29].

The control of each segmental movement was demonstrated by a smooth gradual increase in velocity to midpoint of forward bend, and then a smooth gradual decrease in velocity to the end of the forward bend motion. Overall the phase-plane diagram was bell shaped (Fig. 6a), with a minimal number of local minimum and maximum occurrences (Table 5). No quick out of sagittal plane movements were noticed during the forward bend motion. Inter-segmental movement coordination was also smooth and continuous with a minimal number of local minimum and maximum occurrences (Table 6 and Fig. 6b). These kinematic descriptions are also consistent with clinical definition of typical (normal) forward bending described by Paris [17].

### Altered inter-segmental coordination

aLPR is characterized by either shared movement between the pelvis and lumbar spine, or pelvic dominated motion during the first 1/3rd of the forward bend motion. Motion continues with increased pelvis domination in the second 1/3^rd^ of the motion. In the last 1/3^rd^ of the motion the pattern is dominated by lumbar spine motion (Table 4 and Fig. 5c). Overall this pattern is the reverse of a typical forward bend pattern.

Coupling-angle diagrams revealed sharp increases in the coupling angle in the first 1/3^rd^ of forward bend indicating shared motion between the pelvis and lumbar spine that occurs much earlier than the typical pattern. In the second 1/3^rd^ of forward bend, the coupling angle increased indicating pelvis domination, which also appeared earlier than the typical pattern. In the last 1/3^rd^ of forward bend, the coupling angle decreased indicating a reversed pattern (Fig. 5d).

Data suggested that patterns of lumbopelvic coupling angles are key for identifying aLPR (Table 7 and Fig. 5d). Clinicians seemed to perceive pelvic domination when coupling angle (pelvic-dominated angle) reached 59° (pelvic-lumbar ratio = 1.66:1). At a coupling angle of 59°, timing (relative to the % of motion during forward bending task) that maximizes the agreement between clinical observation and kinematic classification derived from coupling-angle diagram demonstrated transition from lumbar spine domination to pelvis domination in the second 1/3^rd^ of the movement (38%). This slight difference between the clinical definition (shift within first 33% of motion) and kinematic cut point (38% of motion) is likely related to time normalization and averaging data across subjects. However, the lumbopelvic coupling-angle diagram represents what the clinicians observed as altered lumbopelvic rhythm with focus on the amount of pelvis contribution (pelvis domination) in the early phase (first 38%) of the movement.

A limited number of studies exist that describe trunk and pelvic angular motion during a standing trunk forward bend task [20, 21, 39, 40]. In these studies, the researchers investigated ratios of pelvis to lumbar segment motion at discrete points in the movement, and reported means and standard deviations. Although they report differences between healthy and low back pain groups, this approach does not provide continuous information about inter-segment coordination and control. Therefore, this existing body of work cannot fully describe altered lumbopelvic rhythm and pinpoint when transition from lumbar domination to pelvic domination occurred. The only reported approach that focused on continuous lumbar spine and pelvis movement coordination was a study that used lumbopelvic segment angle-angle diagrams to represent typical lumbopelvic rhythm [41]. The finding from this study was similar to our finding in which typical lumbopelvic rhythm demonstrated that the overall diagram was a concave line. The lumbar spine had a greater contribution in the early stage of the motion followed by shared motion between the lumbar and pelvic segments, and the pelvis had a greater contribution in the last stage of the motion. Our approach of using lumbopelvic coupling-angle diagrams provides typical timing (relative to % of movement) for when the transition from lumbar to pelvic domination occurs. This spatial and temporal information can be used to further explain altered lumbopelvic rhythm.

### Altered movement control

Qualitative assessment of segment control during forward bend suggests that the frequency of disruptions, or sudden decreases and increases in angular velocity, in the JUD group were significantly greater than the typical group (Table 5). Coupling-angle diagrams also revealed that the JUD group had a greater number of sudden decoupling instances in inter-segmental coordination than the typical group (Table 6).

The data indicate that the kinematic patterns in phase-plane diagram considered as JUD are best defined as the number of local minimum occurrences equal to or greater than 6 in the lumbar spine segment. Clinicians appear to focus on lumbar spine angular velocity or smoothness of the movement during the standing trunk forward bend (Fig. 6a and c). This kinematic description was consistent with clinical observation of JUD (a sudden deceleration and acceleration). Additionally, quick out of plane movement is best defined as when the pattern momentarily deviates away from sagittal plane in lumbar spine plane angle-angle diagram (Fig. 4). Quick out of plane movements were not frequently demonstrated in our dataset. We found that the occurrence of quick out of plane movement was consistent with disruption in angular velocity in the lumbar spine phase-plane diagram. This suggests that clinical observation of judder based on lumbar spine velocity may be sufficient and observation of quick out of plane motion might not be necessary for determination of judder. To date, no researcher has investigated the primary segment and movement control characteristics that represent JUD during standing trunk forward bend.

The data also indicate that JUD can be quantified as number of local minimum and maximum occurrences equal to or greater than 15 in coupling-angle diagram (Fig. 6b and d). Although the kinematic description using coupling-angle diagram was not matched with clinical observation of JUD, it seemed clinicians’ classification of this aberrant movement pattern is made through particular attention to changes in inter-segment coordination between the pelvis and lumbar spine. Potentially, this kinematic description of judder could be used to refine clinical observation of judder.

Collectively, the findings from our study provide detailed descriptions of temporal and spatial 3-dimensional multi-segmental kinematics of the pelvis and trunk segments for typical and aberrant movements during standing forward bend motion. This information can be used to enhance knowledge and understanding of inter-segment coordination and movement control, and may help refine operational definitions that clinicians use to identify aberrant movement patterns. This information may also be useful for future studies that investigate typical and aberrant movement patterns or are designed to determine the ability of exercise and motor control based therapeutic interventions to alter these patterns.

The findings of this study should be considered in light of the following limitations. Our data interpretation was based on the observations of two experienced orthopedic physical therapists which limits generalizability. Data interpretation may also be influenced by their clinically imposed thresholds of aberrance. These thresholds directly affect the prevalence of typical and aberrant ratings. Our approach to analysis was from an accuracy statistics perspective using maximum agreement to develop thresholds. It is possible that these thresholds or criteria are not the same as those used by other clinicians. We also had a relatively low percentage of mutual agreement between two experienced clinicians when we included only those repetitions where both raters indicated a typical pattern or only one type of aberrant pattern on the same repetition. This was done to ensure that movement patterns we analyzed were clear representations of a typical or aberrant pattern. Our prior work focusing on clinical agreement (clinician’s come to the same overall decision about typical or aberrant pattern for the subject) demonstrated moderate to almost perfect agreement (kappa = 0.46–0.83) [18]. But this does suggest that multiple repetitions are likely necessary for clinical agreement on movement patterns. We also acknowledge the limitations associated with the use of the same data set to develop and test accuracy of the kinematic variables. However, this works serves as a starting point for quantification of aberrant movement patterns and we recognize that further work and analysis is warranted.

## Conclusion

Angle-angle, coupling-angle, and phase-plane diagrams can be used to qualitatively and quantitatively describe 3-dimensional multi-segmental kinematic patterns that represent both typical and aberrant (altered lumbopelvic rhythm, or judder) movements during standing forward bend. Coordination of the movement between the pelvis and lumbar spine can be assessed for presence of altered lumbopelvic rhythm and judder. The lumbar spine segment appears to be the key segment to observe judder. These detailed kinematic descriptions should provide clinicians with direction for identifying aberrant movement patterns. Collectively, these data can be used to help improve understanding of typical and aberrant movement patterns, train clinicians in their clinical observation of typical and aberrant movement patterns and to test the efficacy of interventions to change inter-segmental coordination and control.

## Abbreviations

AA: Angle-angle diagram
aLPR: Altered lumbopelvic rhythm
ASIS: Anterior superior iliac spine
AUC: Area under the receiver operating characteristic curve
CA: Coupling angle diagram
CMC: Coefficient of multiple correlation
FP: Pelvic motion with respect to the femur
FT: Thoracic spine motion with respect to the femur
ISB: International Society of Biomechanics
JUD: Judder
LBP: Low back pain
LMax: Local maximum
LMin: Local minimum
LPR: Lumbopelvic rhythm
LR: Likelihood ratio
LSum: Sum of local minimum and maximum
LT: Thoracic spine motion with respect to the lumbar spine
MCI: Movement coordination impairment
NSLBP: Non-specific low back pain
PABAK: Prevalence-adjusted bias-adjusted kappa
PL: Lumbar spine motion with respect to the pelvis
PP: Phase-plane diagram
PSIS: Posterior superior iliac spine
PT: Combined lumbar-thoracic spine motion with respect to the pelvis
ROC: Receiver operating characteristic curve
